# Supplementary material for: Splice-Junction-Based Mapping of Alternative Isoforms in the Human Proteome
Source: Cell Rep. Author manuscript; Available in PMC 2020 Jan 15. (PMC6961840; doi:10.1016/j.celrep.2019.11.026)
Supplement: 2 [file NIHMS1546469-supplement-2.zip › DF1/PXD010154/Heart/Heart_72_NRAP_ASGDIASDLR.pdf]

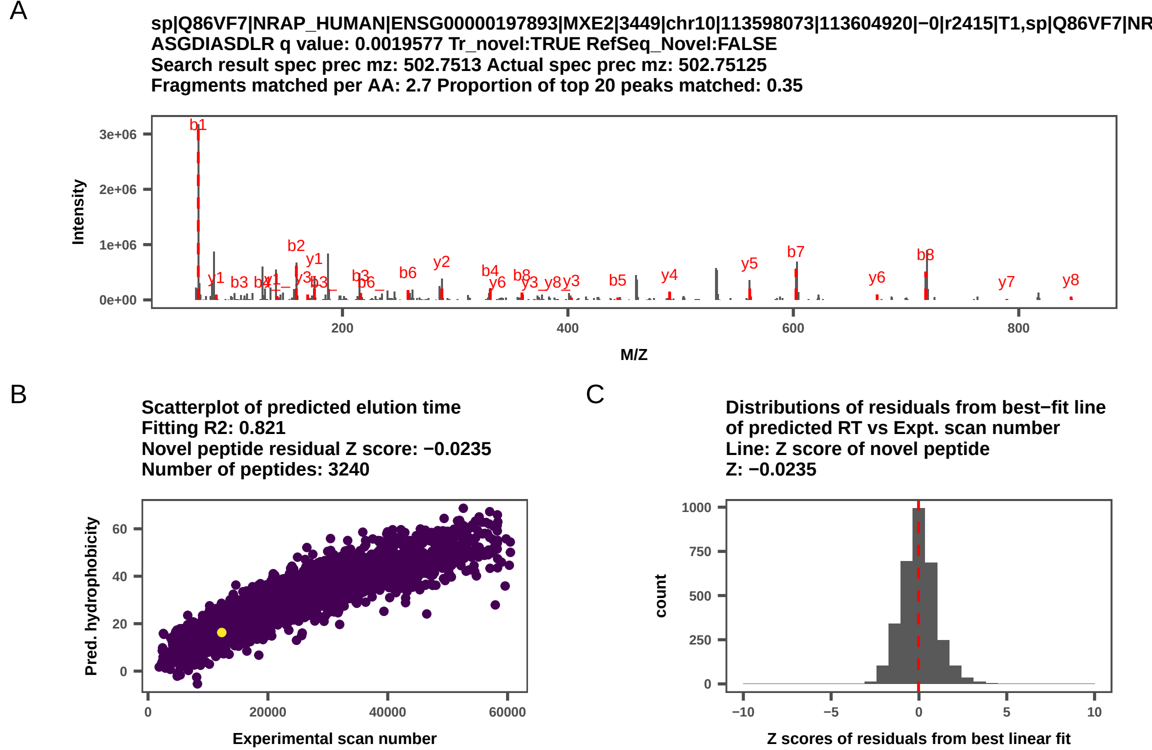
A
sp|Q86VF7|NRAP_HUMAN|ENSG00000197893|MXE2|3449|chr10|113598073|113604920|−0|r2415|T1,sp|Q86VF7|NR
ASGDIASDLR q value: 0.0019577 Tr_novel:TRUE RefSeq_Novel:FALSE
Search result spec prec mz: 502.7513 Actual spec prec mz: 502.75125
Fragments matched per AA: 2.7 Proportion of top 20 peaks matched: 0.35
Intensity
3e+06
2e+06
1e+06
0e+00
b1
b2
y1
b6
y2
b4
b7
y5
b8
y6
y4
b5
y7
y8
200
400
600
800
M/Z
B
Scatterplot of predicted elution time
Fitting R2: 0.821
Novel peptide residual Z score: −0.0235
Number of peptides: 3240
Pred. hydrophobicity
60
40
20
0
0
20000
40000
60000
Experimental scan number
C
Distributions of residuals from best−fit line
of predicted RT vs Expt. scan number
Line: Z score of novel peptide
Z: −0.0235
count
1000
750
500
250
0
−10
−5
0
5
10
Z scores of residuals from best linear fit
